# Popping sound and streaming water-like tinnitus in Cochlin-tomoprotein-confirmed perilymphatic fistula with antecedent barotrauma

**DOI:** 10.3389/fneur.2026.1939108

**Published:** 2026-08-27

**Authors:** Yukihide Maeda, Han Matsuda, Tomoyasu Kitahara, Masafumi Sawada, Ryuichiro Araki, Hiroe Kudo, Kei Sakamoto, Atsuya Takayama, Tetsuo Ikezono

**Affiliations:** 1 Department of Otorhinolaryngology, Saitama Medical University, Iruma-gun, Saitama-Ken, Japan; 2 Medical Education Center, Saitama Medical University, Iruma-gun, Saitama-Ken, Japan

**Keywords:** antecedent traumatic events, barotrauma, Cochlin-tomoprotein, fistula sign, nystagmus, perilymphatic fistula, popping sounds, streaming water-like tinnitus

## Abstract

**Background:**

Perilymphatic fistula (PLF) after barotrauma has been linked to popping sounds, streaming water-like tinnitus, and fistula signs, but objective evidence is limited.

**Aims/objectives:**

To determine whether these features are associated with Cochlin-tomoprotein (CTP)-confirmed PLF after antecedent barotrauma.

**Materials and methods:**

We retrospectively analyzed 149 category 2 (external barotrauma) or category 3 (internal pressure-related barotrauma) cases among 736 patients who underwent CTP testing at 80 Japanese institutions. Frequencies of clinical features were compared by CTP status using Fisher’s exact test.

**Results:**

CTP positivity was 17% in category 2 and 22% in category 3. In category 2, CTP positivity was higher in patients with a popping sound than in those without it (60% vs. 12%; OR, 10.88; 95% CI, 1.63–68.66; *p* = 0.035). In category 3, CTP positivity was higher in patients with streaming water-like tinnitus than in those without it (41% vs. 16%; OR, 3.75; 95% CI, 1.30–10.31; *p* = 0.019) and in patients with nystagmus than in those without it (30% vs. 12%; OR, 3.12; 95% CI, 1.18–8.70; *p* = 0.033). Fistula signs were not associated with CTP positivity.

**Conclusions and significance:**

Symptom profiles differed by barotraumatic mechanism in CTP-confirmed PLF. A popping sound may represent a characteristic clinical clue suggestive of PLF after external barotrauma, whereas streaming water-like tinnitus and nystagmus may better indicate PLF after internal pressure-related events, although the observed associations in this study were exploratory and not adjusted for multiple comparisons. Absence of a fistula sign should not exclude PLF.

## Introduction

Perilymphatic fistula (PLF) is characterized by an abnormal communication between the perilymphatic space of the inner ear and adjacent structures, leading to leakage of perilymph and disturbance in inner ear pressure that can cause inner ear symptoms such as vertigo, hearing loss, tinnitus and aural fullness (1). Fistulas may occur at the round or oval window, fracture sites, micro-fissures, areas of bony labyrinth destruction caused by inflammation, or sites of congenital malformation. Although PLF was initially recognized as a complication of stapes surgery or head trauma, it is now also regarded as a potential cause of acute sensorineural hearing loss and an important differential diagnosis in patients with sudden or fluctuating hearing loss and acute or chronic vertigo (2–5). PLF can be considered a treatable endotype of neurotologic disease because the underlying lesion may be identified and surgically repaired (6–8). Accurate diagnosis is therefore essential for improving patient outcomes.

PLF is classified into four categories based on the antecedent events associated with its onset: category 1, linked to trauma, middle- and/or inner-ear disease, or surgery; category 2, linked to barotrauma caused by antecedent events of external origin; category 3, linked to barotrauma caused by antecedent events of internal origin; and category 4, with no apparent antecedent event (9). A typical history of PLF includes antecedent barotrauma resulting either from external factors, such as diving, air travel, or blast exposure, or from internally generated pressure changes, such as nose blowing, sneezing, or heavy lifting. Careful history taking is particularly important in these patients; therefore, in the present study, we focused on symptoms reported during history taking in category 2 and 3 patients. Patients with these forms of PLF have been empirically reported to present with a popping sound, streaming water-like tinnitus, or a positive fistula sign, but these features have rarely been examined systematically (1, 10, 11). One reason is the longstanding difficulty of establishing a definitive diagnosis. Traditionally, PLF has been diagnosed by identifying perilymph leakage during exploratory tympanotomy, but such intraoperative findings are subjective and observer dependent.

Cochlin-tomoprotein (CTP) is a validated biomarker for the objective diagnosis of PLF. Because CTP is found exclusively in perilymph, its detection by enzyme-linked immunosorbent assay (ELISA) in middle ear lavage fluid enables a definitive diagnosis (12). In the 2016 diagnostic criteria for perilymphatic fistula (PLF) established by the Japanese Ministry of Health, Labor and Welfare, a patient who presents with clinical symptoms of PLF and tests positive for Cochlin-tomoprotein (CTP) can be diagnosed with definite PLF (9). We previously conducted a nationwide multicenter study of patients who underwent CTP testing at over 70 institutions in Japan (9), and since 2022, this assay has been covered by the Japanese public health insurance system. Accordingly, the present study examined whether patients with category 2 or 3 PLF definitively diagnosed by CTP testing exhibit a popping sound, streaming water-like tinnitus, and a positive fistula sign.

## Materials and methods

### Study subjects

In this study, we compiled the medical histories and test results of 736 patients who underwent CTP testing at 80 institutions in Japan between April 2014 and August 2016. A list of participating institutions is provided in the Acknowledgments. In these cases, PLF was suspected on the basis of a history of trauma, middle-ear or inner-ear disease, and/or vestibular symptoms such as vertigo and imbalance, and CTP testing was therefore performed. These cases overlap with those reported in our previous publications (5, 9); however, the present study includes additional cases and presents novel analyses distinct from those previously reported.

Among the 736 cases in total, 149 patients with a documented history of barotrauma resulting from external factors (category 2) or barotrauma caused by internal pressure changes (category 3), based on the classification of the Japanese PLF Study Group shown in Table 1 (5), were selected for analysis.

**Table 1:** Categorization of PLF cases formulated by a Japanese study group (5).

Category 1
Linked to trauma, middle, and/or inner ear diseases, surgeries
(1) a. Direct labyrinthine trauma (stapes luxation, otic capsule fracture, etc.)
b. Other trauma (head injury, body contusion, etc.)
(2) a. Middle or inner ear diseases (cholesteatoma, tumor, anomaly, dehiscence, etc.)
b. Iatrogenic (ear surgeries, medical treatments, etc.)

Category 2
Linked to barotrauma caused by antecedent events of external origin (such as flying or diving)

Category 3
Linked to barotrauma caused by antecedent events of internal origin (such as straining, sneezing, or coughing)

Category 4
Has no apparent antecedent event (idiopathic)
“Spontaneous” should not be used.

The study protocol was approved by the Institutional Review Board of Saitama Medical University (protocol no. 13-086; principal investigator, TI). The study was conducted in accordance with the Declaration of Helsinki.

### CTP testing

At each participating institution, the protocol for collecting middle ear lavage samples for CTP testing was approved by the local ethics committee, and written informed consent was obtained from all patients.

CTP concentrations in middle ear lavage samples were measured at SRL, Inc. (Tokyo, Japan) using an enzyme-linked immunosorbent assay (ELISA) with a polyclonal anti-CTP antibody. Based on a previous report, the cutoff values for diagnosing PLF according to CTP concentration were defined as follows: negative, less than 0.4 ng/mL; intermediate, 0.4 ng/mL to less than 0.8 ng/mL; and positive, 0.8 ng/mL or higher (12). Accordingly, patients with CTP concentrations of 0.8 ng/mL or higher were classified as the CTP-positive group, whereas those with CTP concentrations below 0.8 ng/mL were classified as the CTP-intermediate and negative group.

### Data collection

From the medical records of the 149 category 2 and 3 cases, we extracted age, sex, CTP test results, and the specific precipitating factor for PLF in CTP-positive cases. We also recorded the presence or absence of the following clinical features: popping sound, streaming water-like tinnitus, fistula sign, vestibular symptoms, and nystagmus. Nystagmus was considered present if spontaneous or positional nystagmus was documented at any time after the onset of inner ear symptoms. Hennebert’s sign and the fistula sign were not included in the definition of nystagmus. All participating centers in this study were otolaryngology facilities. The nystagmus was observed in patients who presented to otolaryngology clinics with inner ear symptoms and was therefore thought to be vestibular in origin. Audiometric testing was performed in soundproof rooms at each center. Although vestibular-evoked myogenic potential (VEMP) testing was not performed at all participating centers, among patients with PLF who underwent VEMP testing at our institution (Saitama Medical University), none exhibited enhanced cervical or ocular VEMP responses suggestive of a third mobile window syndrome.

### Statistical analysis

For category 2 and 3 cases, Fisher’s exact test was used to determine whether the frequencies of the five clinical features were higher in the CTP-positive group than in the CTP-intermediate/negative group. All statistical analyses were performed using JMP Pro 16 (SAS Institute, Cary, NC, United States), and statistical significance was defined as *p* < 0.05. All statistical analyses were conducted by a certified biostatistician (RA).

## Results

Among the 149 category 2 and 3 cases (age range, 8–85 years in 136 cases; 67% were male in 138 cases), 41 were category 2 and 108 were category 3. Among CTP-positive category 2 cases, antecedent events were air travel (*n* = 3), scuba diving (*n* = 2), ascent to high altitude (*n* = 1), and removal of an earplug (*n* = 1). Among CTP-positive category 3 cases, precipitating events were nose blowing (*n* = 9), heavy lifting (*n* = 4), childbirth (*n* = 2), defecation (*n* = 2), sniffing (*n* = 1), vomiting (*n* = 1), sneezing (*n* = 1), jogging (*n* = 1), ear clearing by Valsalva maneuver (*n* = 1), abdominal straining (*n* = 1), and clarinet playing (*n* = 1).

The CTP positivity rate was 17% in category 2 cases (7 CTP-positive cases and 34 CTP-intermediate/negative cases) and 22% in category 3 cases (24 and 84 cases, respectively).

**Figure 1:**
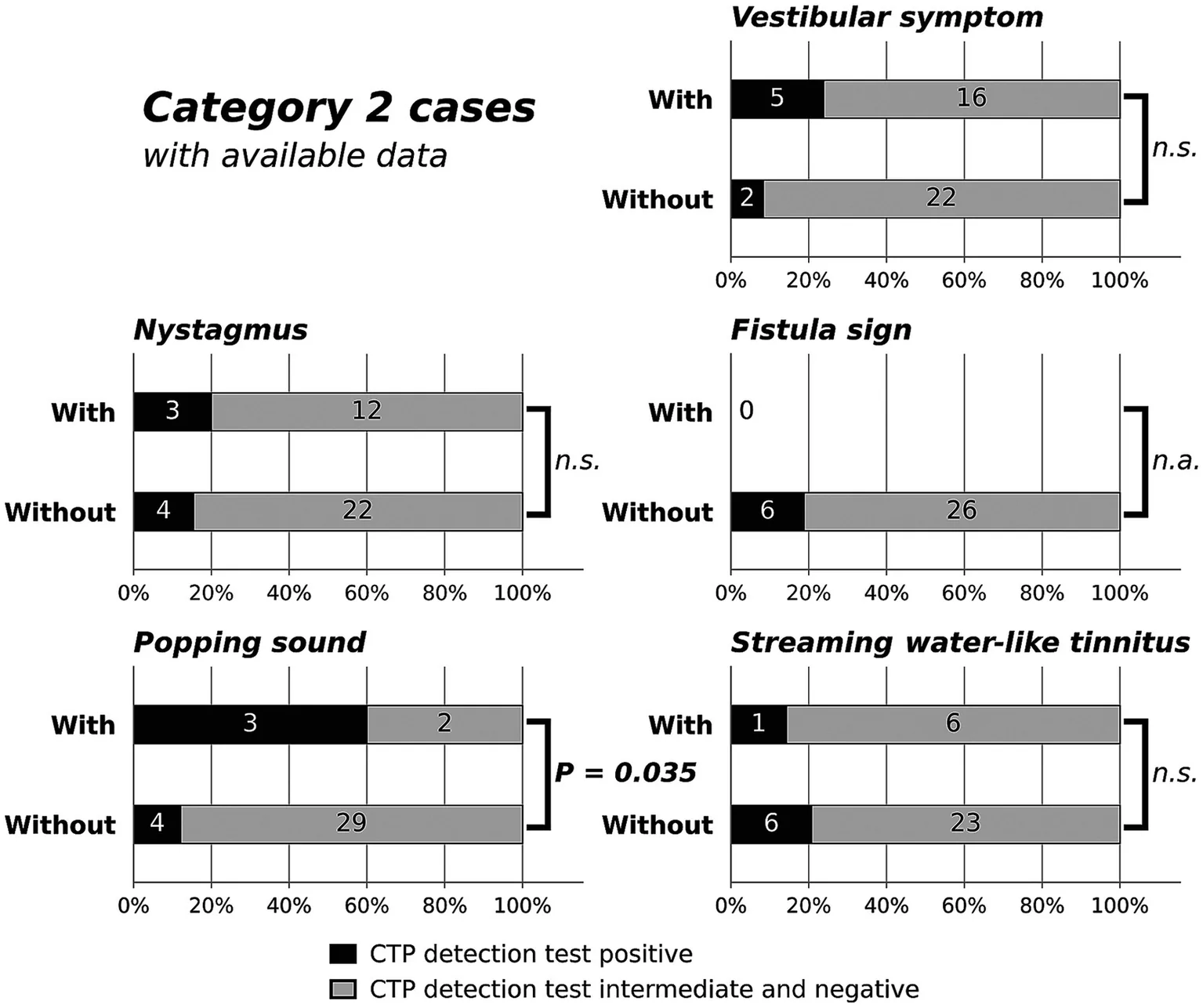
Relationship between clinical features and the rate of Cochlin-tomoprotein (CTP) positivity in category 2 cases. Category 2 cases were defined as those associated with antecedent barotrauma of external origin. The rate of CTP positivity was significantly higher in patients with a popping sound than in those without a popping sound (OR, 10.88; 95% CI, 1.63–68.66; *p* = 0.035, Fisher’s exact test). No significant association was observed between the rate of CTP positivity and the presence or absence of vestibular symptoms, nystagmus, or streaming water-like tinnitus (all *p* > 0.05). No category 2 case showed a positive fistula sign.

Among category 2 cases with available data, CTP positivity was significantly more frequent in patients with a popping sound than in those without a popping sound (60% [3/5] vs. 12% [4/33]; Odds ratio (OR), 10.88; 95% Confidence Interval (95% CI), 1.63–68.66; *p* = 0.035). No significant association was found between CTP positivity and vestibular symptoms (24% vs. 8%; OR, 3.44; 95% CI, 0.57–18.49), nystagmus (20% vs. 15%; OR, 1.38; 95% CI, 0.30–5.79), or streaming water-like tinnitus (14% vs. 21%; OR, 0.64; 95% CI, 0.05–4.54) (all *p* > 0.05). No category 2 case had a positive fistula sign; among fistula sign-negative cases, the CTP positivity rate was 19% (Figure 1).

**Figure 2:**
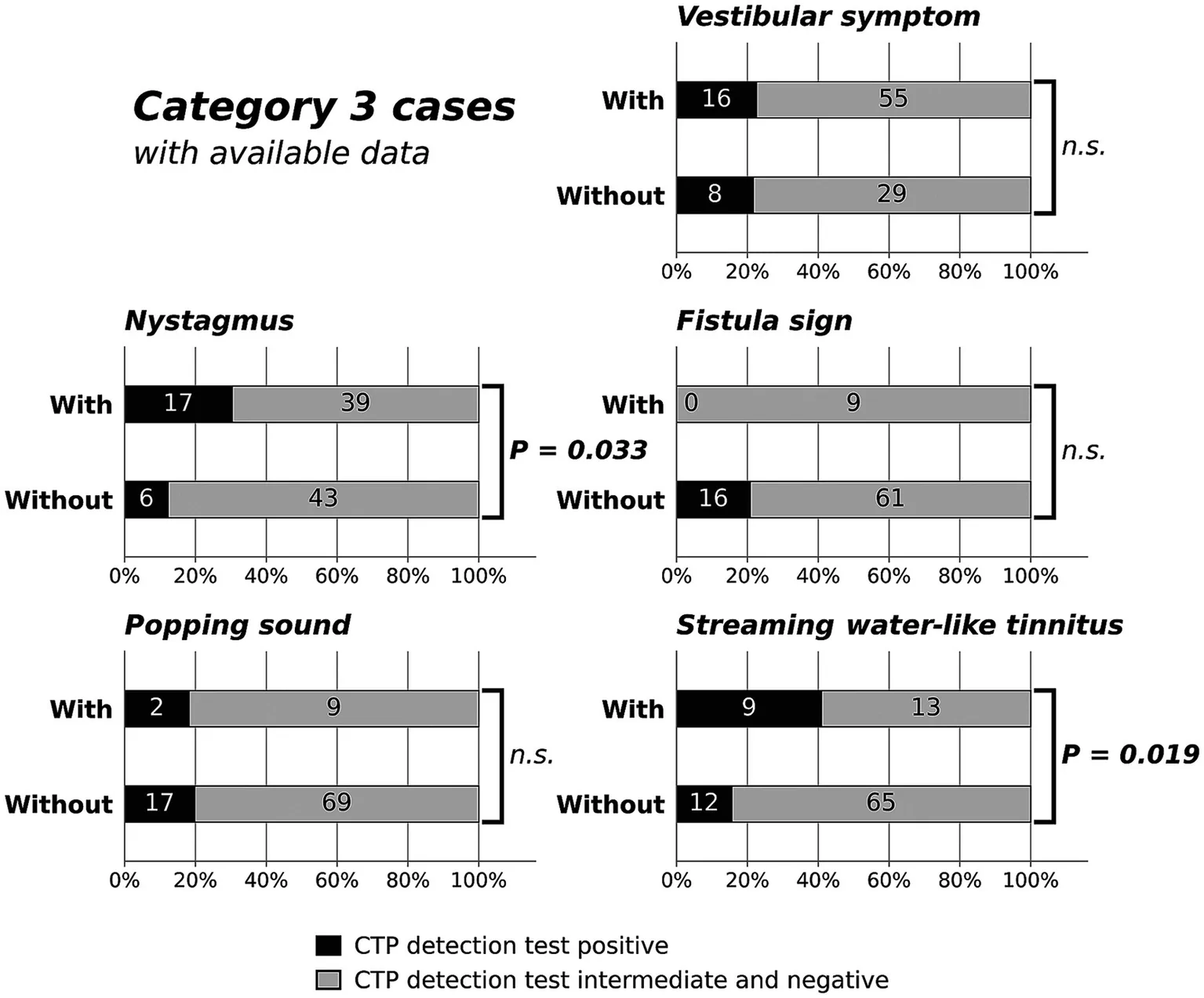
Relationship between clinical features and the rate of Cochlin-tomoprotein (CTP) positivity in category 3 cases. Category 3 cases were defined as those associated with antecedent barotrauma caused by internal pressure changes. The rate of CTP positivity was significantly higher in patients with nystagmus than in those without nystagmus (OR, 3.12; 95% CI, 1.18–8.70; *p* = 0.033, Fisher’s exact test). The rate of CTP positivity was also significantly higher in patients with streaming water-like tinnitus than in those without this symptom (OR, 3.75; 95% CI, 1.30–10.31; *p* = 0.019, Fisher’s exact test). No significant association was observed between the rate of CTP positivity and the presence or absence of vestibular symptoms, a positive fistula sign, or a popping sound (all *p* > 0.05).

Among category 3 cases with available data, CTP positivity was significantly more frequent in patients with nystagmus than in those without nystagmus (30% [17/56] vs. 12% [6/49]; OR, 3.12; 95% CI, 1.18–8.70; *p* = 0.033). CTP positivity was also significantly more frequent in patients with streaming water-like tinnitus than in those without this symptom (41% [9/22] vs. 16% [12/77]; OR, 3.75; 95% CI, 1.30–10.31; *p* = 0.019). No significant association was found between CTP positivity and vestibular symptoms (23% vs. 22%; OR, 1.06; 95% CI, 0.43–2.77), a positive fistula sign (0% vs. 21%; OR, 0.00; 95% CI, 0.00–1.75), or a popping sound (18% vs. 20%; OR, 0.90; 95% CI, 0.18–4.19) (all *p* > 0.05) (Figure 2).

In CTP-positive category 2 cases, the frequencies of nystagmus, a popping sound, and streaming water-like tinnitus were 43, 43, and 14%, respectively. In CTP-positive category 3 cases, the corresponding frequencies were 74, 11, and 43%, respectively. For comparison, among category 4 cases in our dataset (380 cases without antecedent events), the frequencies of a popping sound and streaming water- like tinnitus in CTP-positive cases were low, at 0 and 16%, respectively (Table 2).

**Table 2:** Frequency of nystagmus, popping sound, and streaming water-like tinnitus among Cochlin-tomoprotein (CTP)-positive perilymphatic fistula cases in categories 2, 3, and 4.

PLF category	Nystagmus	Popping sound	Streaming water-like tinnitus
Category 2 (41 cases)	43%	43%	14%
Category 3 (108 cases)	74%	11%	43%
Category 4 (380 cases)	56%	0%	16%

Values are presented as percentages in CTP-positive cases within each category; the numbers in parentheses indicate the total number of cases in that category. Categories 2, 3, and 4 correspond to the case groups with external-origin antecedent events, internal-origin antecedent events, and no antecedent event, respectively.

Among patients in category 2 or 3, 57 had both vestibular symptoms and nystagmus; of these, 16 (28.1%) were CTP-positive.

## Discussion

Traditionally, a typical clinical course of PLF has been described as involving an episode of external or internal barotrauma, followed by the onset of symptoms such as a popping sound or streaming water-like tinnitus (1, 10, 11). This concept was largely empirical and based on the presumed pathophysiology that disruption of inner ear structures leads to leakage of perilymph. In addition, symptoms such as a popping sound and streaming water-like tinnitus are themselves subjective, and the diagnosis of PLF has also traditionally relied on the subjective intraoperative judgment of perilymph leakage during exploratory tympanotomy. In the present study, we provide objective evidence that these symptoms are associated with cases in which the perilymph-specific biomarker CTP was positive, that is, definitively diagnosed PLF. This study was made possible by aggregating 149 cases from 80 institutions across Japan.

Detailed history taking is considered essential for the diagnosis of PLF. A popping sound and streaming water-like tinnitus are history-based findings elicited during the medical interview. In patients presenting with inner ear symptoms, a popping sound was considered present when the patient reported hearing “a sound as though something had popped inside the ear” at or around symptom onset. Similarly, streaming water-like tinnitus was considered present when the patient reported hearing “a sound like water flowing inside the ear.” In the present study, we evaluated the diagnostic utility of these history-based findings by comparing their presence with the results of the CTP test, an objective diagnostic test for PLF. Although some of our patients with PLF might have experienced other history-based symptoms, including high-pitched ringing tinnitus, and some considered hearing loss or vertigo to be the more bothersome symptom, the present study focused on a popping sound and streaming water-like tinnitus that may be useful for the diagnosis of PLF.

Among PLF cases associated with barotrauma, category 2 cases preceded by external barotrauma were associated with a popping sound, whereas category 3 cases preceded by internal pressure-related barotrauma were associated with streaming water-like tinnitus. Thus, the symptoms suggestive of PLF differed between categories 2 and 3. This finding may indicate that the pattern of preceding pressure loading influences clinical presentation. A popping sound may reflect abrupt pressure release or membrane rupture at the moment of fistula formation, whereas streaming water-like tinnitus may reflect a persistent disturbance of perilymph dynamics. In category 3 cases, nystagmus and/or streaming water-like tinnitus were significantly more frequent in CTP-positive patients than in CTP-negative patients. This finding suggests that a disturbance of labyrinthine fluid mechanics caused by internal pressure-related barotrauma may affect both the vestibular and cochlear systems. One possible explanation is that a PLF functions as an abnormal mobile window, allowing pressure-induced displacement of the inner ear fluids to influence both vestibular and cochlear function.

In our dataset, among category 4 cases (those without antecedent barotraumatic events), a popping sound and streaming water-like tinnitus were infrequent, occurring in 0 and 16% of CTP-positive cases, respectively. Therefore, the popping sound and streaming water -like tinnitus observed in category 2 and 3 cases may represent symptoms distinct from nonspecific tinnitus.

Traditionally, the fistula sign has been emphasized as a clinical clue suggestive of PLF (13, 14). In a nationwide survey of CTP testing, fistula signs were observed in 50% of CTP-positive category 1 cases (those linked with trauma, middle, and/or inner ear diseases, surgeries) (9). In the present study, however, no category 2 case had a positive fistula sign, and even in category 3, no significant association was found between a positive fistula sign and CTP-confirmed PLF. Accordingly, the fistula sign does not appear to be highly sensitive, and the absence of this sign should not be used to exclude PLF. By contrast, in category 3, nystagmus was significantly associated with CTP-confirmed PLF related to internal pressure changes, suggesting that nystagmus may be a more objective indicator of vestibular asymmetry.

Because PLF represents an endotype that can be treated surgically with PLF repair, accurate diagnosis is important. The present findings may support more appropriate clinical diagnosis of PLF. Although only about 40% of CTP-positive cases exhibited a popping sound or streaming water-like tinnitus, these symptoms should heighten suspicion for PLF in patients with a clear antecedent barotraumatic event. Ideally, such patients should then undergo objective CTP testing, and PLF repair surgery should be considered when appropriate.

## Limitations

Because the analyses in this study were exploratory, we used an unadjusted screening approach to broadly identify clinical features potentially associated with CTP positivity and did not apply a Bonferroni correction. Therefore, the possibility of false-positive associations due to multiple comparisons should be considered when interpreting the present findings. The observed association between a popping sound and CTP-confirmed PLF in category 2 patients was based on a small number of cases, and the effect estimate had a wide confidence interval. Therefore, this association should be regarded as an exploratory finding that requires confirmation in a larger cohort. This study was retrospective, and assessment of the fistula sign was not standardized across institutions. Because some subgroup analyses included small numbers of patients, these findings should be interpreted with caution. In addition, because the study population was limited to patients with suspected perilymphatic fistula, selection bias may have been present.

## Conclusion

This study provides objective evidence that history-based findings previously regarded as empirical observations are associated with CTP-confirmed PLF. The pattern of relevant clinical findings varied according to the antecedent barotrauma category: a popping sound was associated with CTP-confirmed PLF in category 2 (external barotrauma), whereas streaming water-like tinnitus and nystagmus were associated with CTP-confirmed PLF in category 3 (internal pressure-related barotrauma). These findings underscore the importance of interpreting clinical clues in the context of the mechanism of barotrauma.

## Data Availability

The raw data supporting the conclusions of this article will be made available by the authors, without undue reservation.
